# Leaves of White Beetroot As a New Source of Antioxidant and Anti-Inflammatory Compounds

**DOI:** 10.3390/plants9080944

**Published:** 2020-07-26

**Authors:** Urszula Gawlik-Dziki, Laura Dziki, Jakub Anisiewicz, Ewa Habza-Kowalska, Małgorzata Sikora, Dariusz Dziki

**Affiliations:** 1 Department of Biochemistry and Food Chemistry, University of Life Sciences, 8 Skromna Str., 20-704 Lublin, Poland; laura52757@gmail.com (L.D.); jakub.anisiewicz1@gmail.com (J.A.); ewa.habza1@gmail.com (E.H.-K.); malgorzata.sikora@up.lublin.pl (M.S.); 2 Department of Thermal Technology and Food Process Engineering, University of Life Sciences in Lublin, 31 Głęboka St., 20-612 Lublin, Poland; dariusz.dziki@up.lublin.pl

**Keywords:** beetroot leaves, phenolic compounds, antioxidant activity, lipoxygenase inhibition, xanthine oxidase inhibition

## Abstract

The white beetroot cv. Śnieżna Kula is the first betanin-free beetroot registered in the European Union. The aim of this study was to compare the phenolic acids profile and antioxidant capacity of leaves of white (SK) and red (CC) beetroots and red (LC) and white (BL) Swiss chard growing in Poland. LC leaves were the richest source of total phenolics (16.55 mg GAE/g FW) and phenolic acids (1.81 mg/g FW), while the highest content of flavonoids was determined in CC leaves (1.6 mg QE/g FW). The highest antiradical activity was observed for LC, whereas CC extract exhibited the highest chelating power. BL and CC leaf extracts demonstrated high LOX inhibitory potential (EC_50_ = 53.23 and 56.97 mg FW/mL, respectively). An uncompetitive type of LOX inhibition was obtained for all extracts. SK extracts demonstrated the highest XO inhibitory activity (EC_50_ = 81.04 mg FW/mL). A noncompetitive type of XO inhibition was obtained in both extracts from red leaves (CC and LC), whereas an uncompetitive mode of inhibition was observed in the case of white leaf (SK and LC) extracts. Thus, it can be assumed that the presence of betanin influences the XO inhibition mechanism.

## 1. Introduction

**Figure 1:**
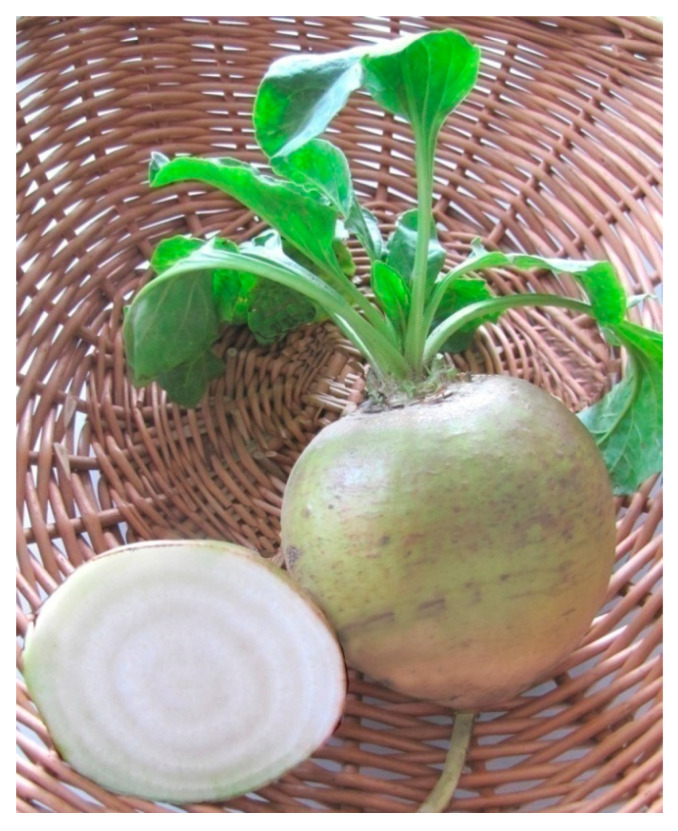
Beetroot “Śnieżna Kula”. Ownership: Torseed S.A.

The red beetroot (*Beta vulgaris* L.) is commonly cultivated and consumed in Poland and other countries of the East. Recently, a new variety of the red beetroot called “Śnieżna Kula” (Eng. “snow ball”) has appeared in the world market. The plant is a result of multiyear work conducted by researchers and produced by a Polish company—TORSEED. Śnieżna Kula is the first betanin—free beetroot registered in the European Union (Figure 1).

The vegetable has retained the taste and aromatic properties of the red beetroot as well as agrotechnical requirements. The white beetroot was created mainly for Italian, French, and English markets, where betanin was withdrawn from diets for babies and children due to its allergenic properties.

The root is the traditionally eaten part of these plants. Whilst most studies assessing the beneficial effect of the red beetroot have been limited to the root, the nutritional and nutraceutical potential of leaves is underestimated. Red beet leaves are consumed in salads with other vegetables worldwide. In Korea, leafy vegetables, including red beet leaf, are commonly used for wrapping cooked rice or meat [1]. In turn, a cultivar of *Beta vulgaris*—i.e., the Swiss chard is commonly cultivated and consumed in Mediterranean countries. It constitutes one of the basic components of the Mediterranean diet, but is also becoming part of other diets. Given the increasing awareness of the potential benefits provided by plant foods, the Swiss chard has recently gained popularity [2].

Food is the main source of principal antioxidants represented by phenolic compounds. They exhibit anti-inflammatory and anticancer properties, which has been well documented [3]. One of the plants with a high level of phenolic compounds are beetroots and Swiss chards, which have been undervalued in terms of their pro-health influence to date [4]. Nevertheless, the prominent properties of beetroot as a promising preventive or therapeutic agent in oxidative stress and inflammatory conditions are reported. In particular, there is ample evidence now that the contribution of beetroot in cardiovascular diseases was likely due to its ability to diminish NO bioavailability and improve endothelial function [4].

Given the high level of phenolic compounds and antioxidant potential of beetroot leaves, inclusion thereof in the diet may bring natural protective effects against so-called diseases of affluence, e.g., cancer or cardiovascular disease and other free radical-related diseases [2].

Disproportion between reactive oxygen species (ROS) and antioxidants is defined as an oxidative stress (OS). Oxygen radicals such as hydroxyl radical, •OH, and nonradical substances, such as hydrogen peroxide H_2_O_2_, are examples of ROS. [5]. Overproduction of reactive species leads to OS, which causes damage to basic biomolecules, e.g., proteins, DNA, and lipids. Additionally, ROS can interfere with intracellular signal transduction processes, which have been implicated in the development of insulin resistance, inflammation, and various lifestyle diseases, for example, cancer, cardiovascular diseases including atherosclerosis, hypertension heart failure, etc. [6,7].

However, ROS play an important role in immune reactions, for example, in combating pathogens [8]. ROS can also be generated in the cells via enzymatic reactions. Free radicals are generated in processes related to the respiratory chain, phagocytosis, prostaglandin synthesis, and in the cytochrome P450 system [9]. The lipoxygenase (LOX) and xanthine oxidase (XO) enzymes are involved in the production of free radicals.

LOX catalyzes incorporation of oxygen to the double bond in polyunsaturated fatty acids, e.g., linoleic acid, oleic acid, and arachidonic acid. This reaction results in the production of hydroperoxides and epoxide. Overexpression of LOX is detected during inflammation and tumorigenesis primarily in immune, epithelial, and tumor cells [10].

XO catalyzes the process of oxidation of hypoxanthine to xanthine and oxidation of xanthine to uric acid. Hydrogen peroxide (H_2_O_2_) exhibiting an affinity for iron is generated as a side effect of these reactions. In the reaction with iron, H_2_O_2_ is converted into hydroxyl radical (•OH). •OH^−^ initiates the generation of subsequent free radicals in reactions with lipids, proteins, and nucleic acids [11]. To the best of our knowledge, there is no study of the antioxidant activity of the white beetroot in the context of the absence of betanin. Moreover, there are limited studies concerning the antioxidant activity of *Beta vulgaris* subspecies *cycla*.

Beetroot leaves (especially young ones) are consumed as an ingredient in salads and used for the preparation of soups. White beetroot is a new and little known plant. In turn, Swiss chard is a leafy vegetable gaining popularity in Poland, but it is still occasionally harvested. A comparison of these plants seems relevant since, despite the differences, they are representatives of the same species. Therefore, the aim of this study was to investigate and compare the nutraceutical potential of leaves of two cultivars of beetroots (*Beta vulgaris* L.): White (Śnieżna Kula Eng. “white ball”) and red (Czerwona Kula, Eng. “red ball”) beetroot and two cultivars of *Beta vulgaris* subsp. *vulgaris*, Cicla-Group and Flavescens-Group; red—Rhubarb Chard and white—Lukullus growing in Poland.

## 2. Results

### 2.1. Analysis of Phenolic Compounds

Plants containing a high amount of phenolic compounds are a good source of antioxidants [6]. This fact prompted the determination of the phenolic acids (PA) profile as well as the content of total phenolics (TPC) and flavonoids (TFC) in the samples. As presented in Table 1, the red chard leaves were the richest sources of phenolic acids (PA) (180.90 mg/100 g FW). Surprisingly, the lowest content of total PA was determined in red beetroot leaves (116.89 mg/100 g FW). The total contents of PA in the case of the white chard and SK leaves were quite similar (152.35 and 141.64 mg/100g FW, respectively). Interestingly, the PA profile of the SK and LC leaves was the same, but the amounts of phenolic acids differed significantly. The difference is mainly related to the salicylic acid content (Table 1).

**Figure 2:**
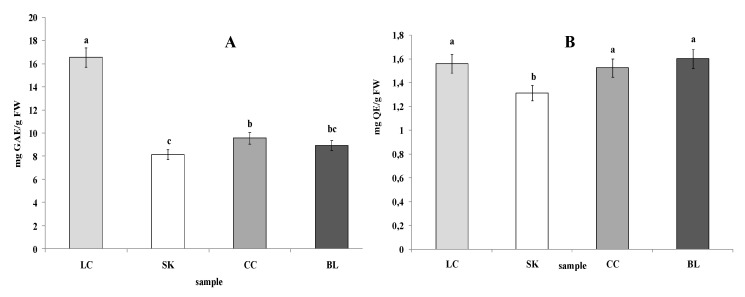
Phenolics (**A**) and total flavonoids content (**B**) in leaves of red chard (LC), white beetroot (SK), red beetroot (CC), and white chard (BL). Results are presented as the mean ± SD; the values designated by the different small letters (a, b, c, d) are significantly different (α = 0.05).

Unexpectedly, the highest TPC was determined in the LC leaves (16.55 mg GAE/g FW). The lowest TPC value was obtained for the SK and BL leaf extract (8.15 and 8.94 mg GAE/g FW, respectively) (Figure 2B). The highest content of TF was determined in the case of CC (1.6 mg QE/g FW) and both chard samples (over 1.5 mg QE/g FW). The lowest flavonoid content (1.31 mg QE/g FW) was determined in the SK samples (Figure 2B).

### 2.2. Total Betalain Content

**Figure 3:**
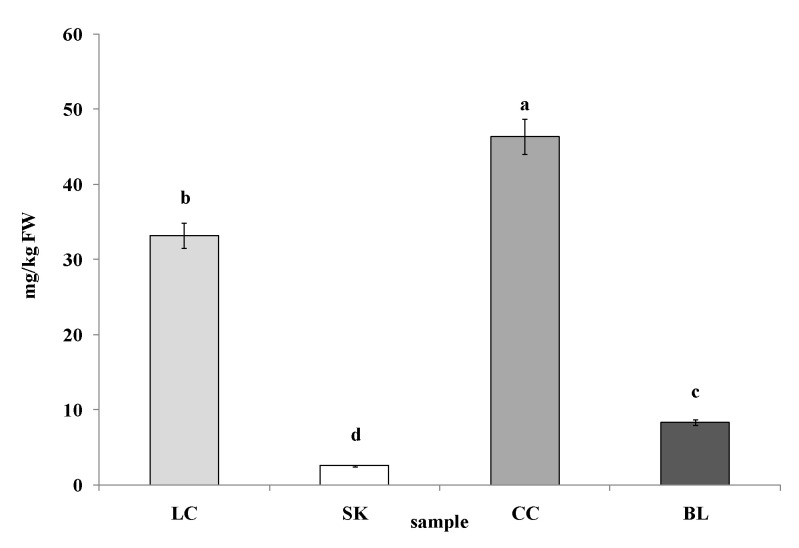
Betalains content in leaves of red chard (LC), white beetroot (SK), red beetroot (CC), and white chard (BL). Results are presented as the mean ± SD; the values designated by the different small letters (a, b, c, d) are significantly different (α = 0.05).

The betalain content in red beetroots has been widely studied; however, there are only sparse data on their content in leaves. As expected, the highest content of betalains expressed as a betanin equivalent was found in the CC leaves (46.42 mg/kg FW). A significantly lower amount of this pigment was determined in the LC sample (33.22 mg/kg FW). The lowest betalains content (2.55 mg/kg FM) was detected in the SK leaves (Figure 3).

### 2.3. Antioxidant Capacity

**Figure 4:**
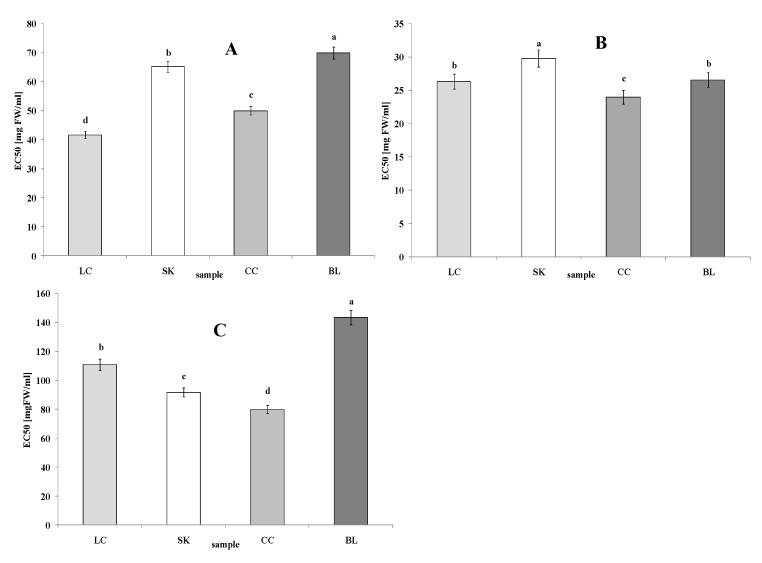
ABTS free radicals scavenging ability. (**A**) Hydroxyl radicals scavenging ability, (**B**) and chelating power (**C**) of extracts from red chard (LC), white beetroot (SK), red beetroot leaves (CC), and white chard (BL). Results are presented as the mean ± SD; the values designated by the different small letters (a, b, c, d) are significantly different (α = 0.05).

In terms of the ability to neutralize ABTS free radicals, the highest activity was noted for LC (EC_50_ = 41.65 mg FW/mL). A slightly lower but statistically significant activity was noticed for the CC sample (EC_50_ = 50.00 mg FW/mL). Extracts from SK and BL showed a similar low activity (EC_50_ = 65.20 and 69.97 mg FW/mL, respectively) (Figure 4A). In turn, the highest ability to neutralize OH• radicals was observed for the LC extracts (EC_50_ = 50.00 mg FW/mL), whereas SK showed the lowest activity (EC_50_ = 29.78 mg FW/mL). LC and BL did not exhibit statistically significant differences (EC_50_ = 26.32 and 26.56 mg FW/mL, respectively) (Figure 4B). Our investigation showed that the CC extract exhibited the highest chelating power (EC_50_ = 79.98 mg FW/mL), whereas lower values were observed for SK and LC (EC_50_ = 91.80 and 111.05 mg FW/mL, respectively). The lowest activity was noticed for BL (EC_50_ = 143.46 mg FW/mL) (Figure 4C). The positive control values (expressed as EC_50_) were 0.103 and 0.036 mg TA/mL for ABTS and OH assays, respectively, and 0.372 mg EDTA/mL for the CHEL assay.

### 2.4. Lipoxygenase Inhibition

**Figure 5:**
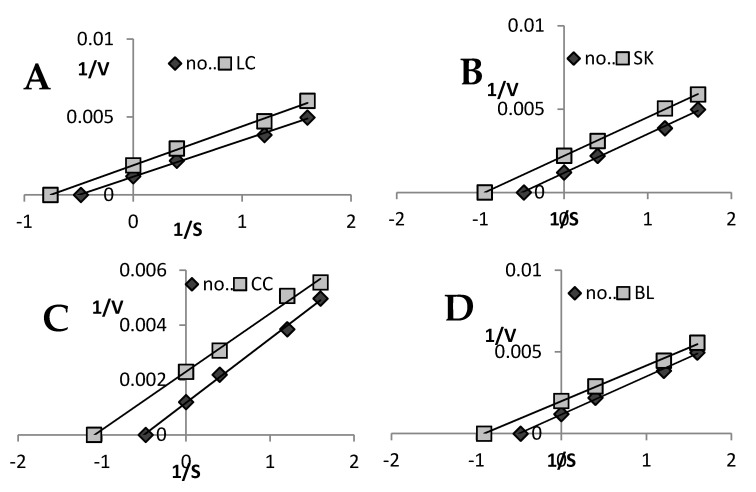
Mode of lipoxygenase inhibition by extracts from red chard (**A**), white beetroot (**B**), red beetroot leaves (**C**), and white chard (**D**). LC—red chard; SK—white beetroot; CC—red beetroot leaves; BL—white chard.

As presented in Table 2, the highest LOX inhibitory potential was found for the BL and CC leaf extracts (EC_50_ = 53.23 and 56.97 mg FW/mL, respectively), whereas the lowest value was determined in the case of the LC extract (78.29 mg FW/mL). As shown in Table 2 and Figure 5, an uncompetitive type of LOX inhibition was determined in all extracts.

### 2.5. Xanthine Oxidase Inhibition

**Figure 6:**
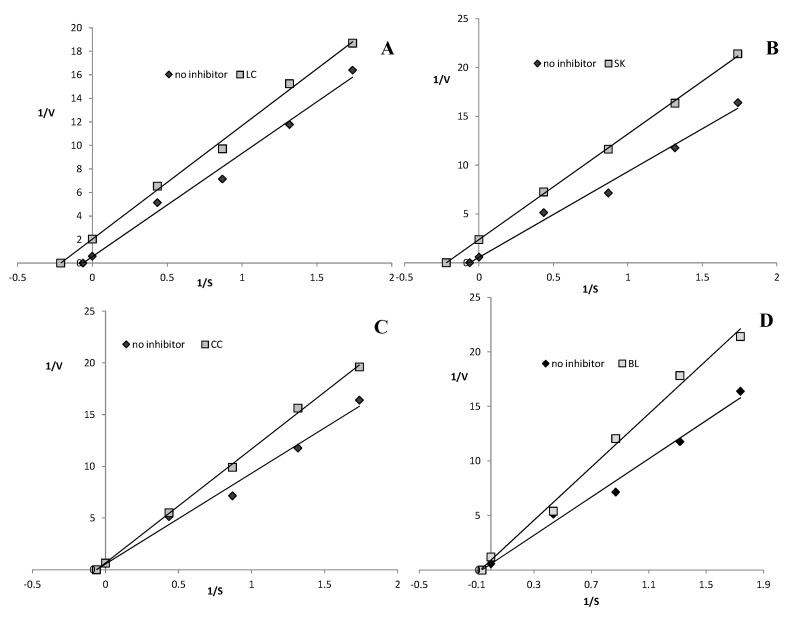
Mode of xanthine oxidase inhibition by extracts from red chard (**A**), white beetroot (**B**), red beetroot leaves (**C**), and white chard (**D**). LC—red chard; SK—white beetroot; CC—red beetroot leaves; BL—white chard.

As presented in Table 3, the highest XO inhibitory potential was found for the SK extract (EC_50_ = 81.04 mg FW/mL), whereas the lowest value was determined in the case of the CC extract (205.09 mg FW/mL). As shown in Table 3 and Figure 6A,C, a noncompetitive type of XO inhibition was obtained in both extracts from the red leaves (CC and LC), whereas an uncompetitive mode of inhibition was noted in the case of the white leaf extracts (SK and LC) (Figure 6B,D). Based on these observations, it can be assumed that the presence of betanin influences the XO inhibition mechanism; however, confirmation of this thesis requires further research.

## 3. Discussion

Almost all the plant-derived foods, which constitute a large part of the human diet, are rich in phenolic acids. The average intake of phenolic acids has been reported to reach 200 mg/day depending on the diet habits and preferences [12].

As presented in Table 1, the red chard leaves were the richest sources of phenolic acids (1.81 mg/g FW). Unexpectedly, the lowest content of total PA was determined in the leaves of red beetroot (116.89 mg/100 g FW). The total PA contents in the white chard and SK leaves were quite similar. Interestingly, the PA profile of the SK and LC leaves was the same, but the amounts of phenolic acids differed significantly. Protocatechuic, *p*-hydroxybezoic, caffeic, syryngic, ferulic, *p*-coumaric, synaptic, and salicylic acids were identified. The results are in accordance with those obtained in earlier investigations [12,13]. Gallic, ferulic, chlorogenic, caffeic, vanillic, syringic, and ellagic acid were identified in roots and stems of red beet [13]. The water extract of chard leaves had higher levels of vanillic, caffeic, and ellagic acids (85.91, 28.45, and 12.58 ppm, respectively), while the methanolic extract of chard leaves was richer in vanillic, salicillic, and protocatechuic acids (103.32, 62.32, 22.6, and 12.72 ppm, respectively). In contrast, water and methanolic extracts of beetroot leaves was higher in vanillic, ellagic, and protocatechuic acids [14].

The total concentration of phenolic compounds in beetroots and Swiss chard has been widely studied; however, investigation results differ significantly. As shown by Pyo et al. [2], the total content of phenolics (red; 128.1 mg/100 g FW, white; 101.5 mg/100 g FW) was substantially higher in leaf extracts than in stem extracts (red; 29.7 mg/100 g FW, white; 23.2/100 g FW). Sacan and Yanardag [15] demonstrated 11.88 ± 1.46 µg epicatechin equivalents of phenolics in 1 mg of water extracts of chard. Ninfali and Angelino [16] showed that the TPC content in *Beta vulgaris cicla* leaves was 11.12 mg/g DW, while the average flavonoid level was 7.92 mg/g DW. For comparison, *Beta vulgaris rubra* leaves contained 12.76 mg TPC per g DW and 11.64 mg flavonoids per g DW. However, it should be noted that the authors did not provide accurate systematic data or the extraction method; hence, comparison of the results is difficult. Noteworthy, the content of phenolics is significantly influenced by the cultivation conditions and the methodology used.

Flavonoids are well-known natural antioxidants. Therefore, dietary intake of foods rich in flavonoids—has been suggested to contribute to protection from free radical damage. Our study indicated that all analyzed materials contained comparable amounts of flavonoids. In the paper by Sacan and Yanardag [15], the methanol-water (8:2) extracts of green and yellow Swiss chard cultivars were compared in terms of their flavonoid composition and content. The total flavonoid contents were 2.76 and 1.26 mg/g of fresh weight, respectively. A phenolic fraction obtained from the ethanol-water (4:6) Swiss chard extract was analyzed and 2″-xylosylvitexin, isorhamnetin 3-gentiobioside, and vitexin 2O-rhamnoside were identified as major components, whereas rutin, quercetin 7-glucuronide, isorhamnetin, and apigenin 7-rutinoside were minor compounds [16]. Similarly, Chandra et al. [17] reported flavonoid content of 11.08 mg QE/g DW in chard, which is generally consistent with our results. In turn, significantly lower values were obtained by Zein, Hashish, and Ismaiel [14], who analyzed the phenolic contents in white chard and beetroot leaves. As demonstrated by the authors, the total flavonoids content was on average 0.19 and 0.48 mg/g FW, respectively.

Due to their antioxidant activity, betalains protect from free radical-related disorders. Therefore, regular consumption of betalain-rich products or foods colored with betalains, which are considered to be safe natural food dyes, can exert a positive effect on the consumer’s organism. Betanin which is listed as food additive E162, is commonly used in a multiple of food products. Red beetroots were found to be a rich source of betanin (about 300–600 mg/kg) [18]. The results obtained in our laboratory are generally in agreement with those reported by Kugler et al. [19]. As shown in their study, red-purple petioles contained 50.6 mg of betalains per kg of FW and yellow stems—49.7 mg/kg of FW. In turn, the lowest values were obtained in the case of yellow-orange Swiss chard petioles—33.6 mg/kg FW. It worth mentioning that the leaves were analyzed in our study. As reported by Ali et al. [20] the highest content of betacyanin was found in the vein in red beet (271.61 μg betanin equivalents g^−1^ FW), while green amaranth exhibited the lowest content (17.35 μg betanin equivalents g^−1^ FW) under 12 and 24 h photoperiods, respectively.

Plants usually contain mixtures of phenolic compounds. Therefore, their interactions must be borne in mind when discussing the antioxidant activity of phenolics. Many researches have tried to determine the antioxidant potential of a given food based on its total phenolics content and have failed in most cases due to the differences between the theoretical and the calculated antioxidant activity of the analyzed product. Red beetroot leaves are a good source of natural antioxidants. In these leaves ROS induce the synthesis of betacyanin, which can neutralize free radicals and reduce damage caused by bacterial infection and wounding [1].

Antioxidant properties, especially antiradical activities, are very important due to the harmful effect of free radicals in biological systems, including food. A commonly used method to evaluate antiradical activities is the model of scavenging stable ABTS radicals [21].

In our study, the highest antiradical activity was observed for LC (EC_50_ = 41.65 mg FW/mL). Extracts from SK and BL showed similarly low activity (EC_50_ = 65.20 and 69.97 mg FW/mL, respectively) (Figure 4A), probably it is a consequence of a lack of betanin.

Sacan and Yanardag [15] found that the EC_50_ values of the ABTS radical scavenging activity in chard extracts and rutin were 1093.14 ± 52.02 and 113.70 ± 3.24 µg/mL, respectively. As shown by Burri et al. [22], the antioxidant potential of beetroot leaves (measured as ABTS radical scavenging activity) was on average from 8.4 to 10.0 mmol TEAC/100 g DW) [22].

The OH• radical is a highly reactive oxygen species which is commonly formed in vivo and can attack many cell macromolecules, including nucleic acids, lipids, and proteins. It is involved in inflammation-related diseases, e.g., chronic inflammation, neurodegeneration, and cancer [5]. Our results demonstrated highly that the investigated plants exhibited a high ability to neutralize OH• radicals. The highest activity was observed for the LC extracts (EC_50_ = 50.00 mg FW/mL), whereas SK showed the lowest activity (EC_50_ = 29.78 mg FW/mL) (Figure 4B). Studies reported by other researchers confirmed the antiradical potential of beetroots and chard leaves. As demonstrated by Sacan and Yanardag [15], chard extract neutralized OH• radicals at a level of 12.03 ± 1.37% at 0.02 mg/mL and 20.24 ± 6.28% at 0.06 mg/mL. Trolox and BHA exhibited good antiradical activity of 54.31 ± 4.30% and 52.00 ± 3.43% at a concentration of 0.06 mg/mL, respectively. The OH• radical scavenging activity of water extract (20.24 ± 6.28%) was nearly equal to that of ascorbic acid (26.47 ± 2.86%) with EC_50_ values of the water extract (0.158 ± 0.041 mg/mL) lower than that of Trolox (46.76 ± 4.17 µg/mL), ascorbic acid (116.04 ± 4.75 µg/mL), and BHA (51.54 ± 2.24 µg/mL).

Due to the susceptibility of membrane phospholipids to oxidative attack, the process of lipid peroxidation after iron overload has been widely investigated [23]. However, to the best of our knowledge, there are no data on the chelating properties of *Beta vulgaris* leaves. Our investigation showed that the CC extract exhibited the highest chelating power (EC_50_ = 79.98 mg FW/mL), whereas the lowest activity was noticed for BL (EC_50_ = 143.46 mg FW/mL) (Figure 4C). Hydroxyl radical, i.e., the most reactive free radical in vivo, is generated in the reaction of O_2_^•−^ with H_2_O_2_ in the presence of Fe^2+^ or Cu^+^. This process is known as the Fenton reaction [9]. Therefore, the ability of phenolics to chelate strongly prooxidant metal ions such as Cu or Fe contributes to their antioxidant properties. Many plant flavonoids are able to form stable metal complexes through their multiple OH groups and the carbonyl moiety [24,25].

Lipoxygenase (LOX) are enzymes involved in conversion of arachidonic acid to leukotrienes and prostaglandins, which are inflammatory mediators. Several studies indicate a correlation between 5-LOX expression and viability of cancer cells, proliferation, cell migration, invasion, metastasis, and apoptosis pathway induction [26]. The LOX-inhibitory potential of plant extracts and pure chemicals is well known and extensively studied [27,28]. However, to the best of our knowledge, no data on the anti-LOX activity and inhibition mechanisms of *Beta vulgaris* subspecies *cycla* and white beetroot leaves (SK) have been reported so far. As presented in Table 2, the highest LOX inhibitory potential was found for the BL and CC leaf extracts (EC_50_ = 53.23 mg FW/mL and 56.97 mg FW/mL, respectively), whereas the lowest value was determined in the case of the LC extract (78.29 mg FW/mL). The uncompetitive type of LOX inhibition was detected in all cases. As demonstrated by Miguel [29], betanidin and betanin were able to inhibit soybean LOX, and this activity was higher than that of catechin. The anti-inflammatory effects of betalains were evidenced in a study conducted by Vidal et al. [30]. In addition to suppression of COX-2 synthesis, betanidin extracted from beetroot inhibited LOX in a dose-dependent manner [4]. However, our results clearly show that betanin does not play a major role in the LOX-inhibitory potential of the tested extracts. This was clearly visible in the SK and BL samples, which exhibited higher inhibitory potential than LC (Table 2). Although the ability of plant extracts to inhibit LOX is widely documented in the literature [26,27,28] to the best of our knowledge, there are no such studies on beetroot and chard leaves.

Dietary polyphenols are able to reduce uric acid synthesis via xanthine oxidase blocking, suppress renal urate reabsorption, and ameliorate uric acid secretion [31].

As reported by Vulić et al. [32], beetroot pomace extract induced XO inhibition in rat liver homogenates due to the presence of a high amount of phenolic compounds and betalain. However, to the best of our knowledge, there is no similar research of beetroot leaf extracts in the recent literature. The highest XO inhibitory potential was found for the SK leaves extract (EC_50_ = 81.04 mg FW/mL), whereas the lowest value was determined in the case of the CC extract (205.09 mg FW/mL) (Table 3). A noncompetitive type of XO inhibition was detected in both extracts from the red leaves, whereas an uncompetitive mode of inhibition was observed in the case of the white leaf extracts. Thus, it can be assumed that the presence of betanin influences the XO inhibition mechanism.

Medicinal plants are rich sources of phytochemicals with XO-inhibitory activity. Kong et al. [33] investigated 122 traditional Chinese medicinal plants used for treatment of gout and other hyperuricemia-related diseases. Among 122 methanol extracts from these plants, 69 exhibited inhibitory activity at 100 mg/mL, with 29 exerting more than 50% inhibition. In turn, Chen et al. [34] reported higher XO inhibitory activities in extracts from *Koelreuteria henryi*, *Prunus campanulata*, and *Rhodiola rosea*. However, data on XO inhibitors derived from common food are limited. Dew et al. [35] found potent XO inhibitors in black tea, and juices from cranberry and purple grape.

## 4. Materials and Methods

### 4.1. Chemicals

Xantine oxidase (E.C. 1.17.3.2), xanthine, soybean lipoxygenase (E.C. 1.13.11), linoleic acid, ABTS (2,2′-azino-bis(3-ethylbenzothiazoline-6-sulfonic acid), sodium salicylate, quercetin, gallic acid, p-hydroxybenzoic acid, vanillic acid, p-coumaric acid, salicylic acid, and ferulic acid were purchased from Sigma-Aldrich (Poznan, Poland). All other chemicals were of analytical grade.

### 4.2. Plant Material and Extract Preparations

The Growth of Plant Samples. Seeds for beetroot (*Beta vulgaris* L. “Czerwona Kula” (CC) and “Śnieżna Kula”(SK)) and *Beta vulgaris* subsp. *vulgaris*, *Cicla*-Group and *Flavescens*-Group; red—Rhubarb Chard (LC) tested in this study were provided by the Torseed SA corporation (Toruń, Poland). *Beta vulgaris* subsp. *vulgaris*, *Cicla*-Group, and *Flavescens*-Group white—Lukullus (BL) was purchased in PNOS Ożarów Mazowiecki (Poland). The plants were cultivated from April to June of 2019 on an experimental farm belonging to the University of Life Sciences in Lublin, Poland. The soil was characterized by a very low level of phosphorus, potassium, and magnesium content, mean content of humus, and was acidic in reaction. The leaves of all species were collected after 60 days of vegetations, moisture content was 76.2%.

Each sample was fresh, previously cleaned, and washed with distilled water, and ground chard and beetroot leaves (2 g) were extracted by mixing (using a rotator Bio RS-24, Józefów near Otwock, Poland) with 15 mL of 1:1 methanol/water (*v*/*v*) and centrifuged at 10,000 rpm for 15 min [36]. Extraction was repeated twice, the supernatants were combined.

### 4.3. Total Phenolics (TPC) Estimation

Total phenolics content was estimated according to the Folin–Ciocalteau method [37]. A 10 µL of the extract was mixed with 10 µL of H_2_O, 40 µL of Folin reagent (1:5 H_2_O), and after 3 min with 200 µL of 10% Na_2_CO_3_. After 30 min, the absorbance of mixture was measured at a wavelength of 725 nm. TPC was expressed as gallic acid equivalents (GAE) per gram of fresh weight (FW) on the basis of a standard curve of gallic acid.

### 4.4. Total Flavonoids (TFC) Estimation

Total flavonoids content (TFC) was estimated according to Bahorun et al. [38]. One hundred microlitters of sample was mixed with 100 μL 2 g/100 AlCl_3_ × 6H_2_O. After 10 min, absorbance at 430 nm was measured. TFC was expressed as the quercetin (QE) equivalent (mg/g FW) on the basis of a standard curve of quercetin.

### 4.5. Phenolic Acids (PA) Profile

Samples were analyzed with a Shimadzu Prominence Auto Sampler HPLC (SIL-20A; Shimadzu, Kyoto, Japan), equipped with Shimadzu LC-20AT reciprocating pumps connected to a DGU 20A5 degasser with a CBM 20A integrator, SPD-M20A diode array detector, and LC solution 1.22 SP1 software (Shimadzu).

The column used was a 250 × 4.6 mm id, 5 μm Varian ChromSep C18 SS, with a guard column ChromSpher 5 C18 SS 10 × 2 mm at 40 °C. The mobile phase consisted of 4.5% acetic acid (solvent A) and 50% acetonitrile (solvent B) at a flow rate of 0.8 mL/min. At the end of the gradient, the column was washed with 50% acetonitrile and equilibrated to the initial condition for 10 min. Quantitative determinations were performed with the external standard calculation, using calibration curves. The gradient elution was used as follows: 0 min 92% A, 30 min 70% A, 45 min 60%, 80 min 61% A, 82 min 0% A, 85 min 0% A, 86 min 92% A, 90 min 92% A [39]. Identification of PA was conducted by comparing retention time and UV absorption spectra with those of pure chemical patterns. The chromatography peaks were confirmed by comparing the retention times with those of reference standards and by the DAD spectra (200 to 500 nm).

### 4.6. Quantitation of Betalain Content

The total betalains content was determined according to Nillson [40] and was expressed as betanin equivalent (BE) per kg of FW.

### 4.7. Antioxidant Assay

#### 4.7.1. Ability to Scavenge ABTS Radicals

The experiments were carried out using the ABTS decolorization assay [41]. The affinity of test material to quench the ABTS free radical was evaluated according to the following equation:

scavenging% = [(A_C_−A_A_)/A_C_)] × 100, (1)

where A_C_ is the absorbance of the control and A_A_ is the absorbance of the sample.

The scavenging activity was expressed as EC_50_—extract concentration providing 50% of activity based on a dose-dependent mode of action. To determine EC_50_ extracts concentrations ranging from 20 to 50 mg FW/mL were used. As a positive control trolox (TA) was used.

#### 4.7.2. Ability to Scavenge the Hydroxyl (OH•) Radicals

The OH• scavenging ability was performed according to Su et al. [42]. The scavenging activity was calculated using the following equation:

scavenging rate% = [1−(A_1_−A_2_)/Ac] × 100, (2)

where Ac is the absorbance of the control (without tested sample), A_1_ is the absorbance of the tested sample addition, and A_2_ is the absorbance without sodium salicylate.

The scavenging activity was expressed as EC_50_—extract concentration providing 50% of activity based on a dose-dependent mode of action. To determine EC_50_ extracts concentrations ranging from 20 to 50 mg FW/mL were used. As a positive control trolox (TA) was used.

#### 4.7.3. Metal Chelating Activity (CHP)

Chelating power was determined by the method of Guo et al. [43]. The activity was calculated according to the formula:

% inhibition = [1 − (As/Ac)] × 100,(3)

where Ac is the absorbance of the control and As is the absorbance of the sample.

The chelating activity was expressed as EC_50_—extract concentration providing 50% of activity based on a dose-dependent mode of action. To determine EC_50_ extracts concentrations ranging from 20 to 50 mg FW/mL were used. As a positive control ethylenediaminetetraacetic acid (EDTA) was used.

### 4.8. Inhibition of Lipoxygenase Activity (LOXI)

Inhibition of soybean 15-LOX is generally regarded as a predictive for inhibition of the mammalian enzyme [44]. The LOX activity was determined spectrophotometrically at 20 °C by measuring the increase of absorbance at 234 nm over a 2 min period [45]. One unit of the LOX activity [U] was defined as an increase of absorbance by 0.001 after 1 min at λ = 234 nm.

The LOXI activity was expressed as EC_50_—extract concentration providing 50% of activity based on a dose-dependent mode of action.

The mode of inhibition on the enzyme was performed using the Lineweaver–Burk plot (at EC_50_ extract concentration).

### 4.9. Inhibition of Xanthine Oxidase Activity (XOI)

XOI activities with xanthine as a substrate were estimated according to Sweeney et al. [46]. One unit of the XO activity [U] was defined as an increase of absorbance by 0.001 after 1 min at λ = 295 nm.

The XOI activity was expressed as EC_50_—extract concentration providing 50% of activity based on a dose-dependent mode of action.

The mode of inhibition on the enzyme was performed using the Lineweaver–Burk plot (at EC_50_ extract concentration).

### 4.10. Statistical Analysis

Experiments were performed in four repetitions. The one-way analysis of variance and Tukey’s test were used for data analysis with a significance level of α = 0.05.

## 5. Conclusions

The white beetroot is a new plant gaining popularity especially in Eastern Europe. Beetroot leaves are an underestimated part of the plant. As shown in this paper, SK leaves have a multidirectional antioxidant activity. They contain significant amounts of phenolic acids, and the content of flavonoids and total phenolic compounds is slightly lower than that of chards. Particularly noteworthy is the fact that SK leaves are rich sources of xanthine oxidase inhibitors and compounds capable of chelating transition metal ions. Therefore, they may be a valuable component of the diet of patients suffering from hyperuricemic disorder; however, confirmation of this thesis requires further research involving determination of the bioavailability of biologically active compounds.

## Figures and Tables

**Table 1:** Phenolic acids content in leaves of white beetroot (SK), beetroot (CC), white chard (BL), and red chard (LC) [mg/100 g FW].

Phenolic Acid	Red Chard (LC)	White Beetroot (SK)	Red Beetroot (CC)	White Chard (BL)
Protocatechuic	2.83 ± 0.03 ^a^ *	1.20 ± 0.00 ^c^ *	1.72 ± 0.05 ^b^ *	nd
*p*-OH benzoic	2.26 ± 0.06 ^a^	1.21 ± 0.01 ^b^	0.96 ± 0.00 ^c^	1.37 ± 0.01 ^b^ *
Caffeic	2.11 ± 0.01 ^a^	1.79 ± 0.01 ^b^	nd	nd
Syryngic	1.45 ± 0.01 ^b^	1.36 ± 0.01 ^c^	1.74 ± 0.06 ^a^	1.34 ± 0.01 ^c^
Vanilic	1.43 ± 0.02 ^a^	1.36 ± 0.00 ^a^	nd	nd
*p*-coumaric	1.40 ± 0.00 ^a^	1.32 ± 0.00 ^a^	nd	nd
Ferulic	5.32 ± 0.17 ^a^	2.73 ± 0.05 ^c^	4.72 ± 0.21 ^b^	2.81 ± 0.04 ^c^
Synapic	7.92 ± 0.45 ^c^	23.23 ± 1.29 ^a^	14.90 ± 1.16 ^b^	4.59 ± 0.09 ^d^
Salicylic	159.01 ± 9.98 ^a^	108.64 ± 6.23 ^c^	94.56 ± 7.60 ^d^	142.24 ± 9.91 ^b^
Sum	180.90 ^a^	141.64 ^c^	116.89 ^d^	152.35 ^b^

* The values are expressed as the mean ± SD; means with different letter superscripts (a–d) in the rows are significantly different (α = 0.05).

**Table 2:** EC_50_ value, Ki value, Vmax value, and mode of lipoxygenase inhibition by extracts from leaves of white beetroot (SK), beetroot (CC), white chard (BL), and red chard (LC) (*n* = 9).

Plant	Mode of Inhibition	EC_50_ [mg FW/mL]	Ki [mg FW/mL]	Vmax [ΔAU/min]
White beetroot	uncompetitive	72.29 ± 0.85 ^b^	72.01 ± 2.11 ^b^	416.66 ± 8.32 ^b^
Red beetroot	uncompetitive	56.97 ± 1.05 ^c^	55.99 ± 1.21 ^c^	412.58 ± 7.25 ^c^
White chard	uncompetitive	53.23 ± 2.23 ^d^	49.13 ± 1.02 ^d^	400.02 ± 5.55 ^d^
Red chard	uncompetitive	78.29 ± 1.13 ^a^	78.61 ± 3.22 ^a^	434.32 ± 6.25 ^a^

* The values are expressed as the mean ± SD; means with different letter superscripts (a–d) in the columns are significantly different (α = 0.05).

**Table 3:** EC_50_ value, Ki value, Vmax value, and mode of xanthine oxidase inhibition by extracts from leaves of white beetroot (SK), beetroot (CC), white chard (BL), and red chard (LC) (*n* = 9).

Plant	Mode of Inhibition	EC_50_ [mg FW/mL]	Ki [mg FW/mL]	Vmax [ΔAU/min]
White beetroot	uncompetitive	81.04 ± 2.22 ^d^	24.62 ± 0.14 ^d^	0.42 ± 0.01 ^c^
Red beetroot	noncompetitive	205.09 ± 4.58 ^a^	1020.48 ± 13.58 ^a^	1.5 ± 0.02 ^a^
White chard	noncompetitive	147.28 ± 5.32 ^b^	229.71 ± 8.23 ^b^	1.11 ± 0.4 ^b^
Red chard	uncompetitive	105.32 ± 7.41 ^c^	39.156 ± 1.54 ^c^	0.49 ± 0.01 ^c^

* The values are expressed as the mean ± SD; means with different letter superscripts (a–d) in the columns are significantly different (α = 0.05).
